# The mediating effect of social support on the relationship between perceived stress and quality of life among shidu parents in China

**DOI:** 10.1186/s12955-021-01726-8

**Published:** 2021-03-22

**Authors:** Cong Wang, Shuang Lin, Yanni Ma, Yang Wang

**Affiliations:** Department of Social Medicine, School of Public Health, China Medical University, No. 77 Puhe Road, Shenyang North New Area, Shenyang, 110122 China

**Keywords:** Shidu parents, Quality of life, Perceived stress, Social support

## Abstract

**Background:**

“Loss-of-only-child family” refers to the family in which the only child died and the mother has passed her child-bearing age. The parents who are unable to reproduce or do not foster other children are known as “shidu parents” in China. This study aimed to estimate the quality of life (QOL) and the mediating role of social support between perceived stress and QOL in Chinese shidu parents.

**Methods:**

502 shidu parents were recruited in Shenyang city. Shidu parents were asked to complete a questionnaire including the MOS item short from health survey (SF-36), the perceived stress scale-10 (PSS-10) and the functional social support questionnaire (FSSQ). Hierarchical linear regression was performed to assess the associations among perceived stress, social support and QOL. Asymptotic and resampling strategies were used to explore the mediating role of social support.

**Results:**

The mean score of PCS and MCS was 64.83 ± 22.66 and 59.36 ± 21.83, respectively. Perceived stress was found to be negatively associated with both PCS (β =  − 0.21, *p* < 0.001) and MCS (β =  − 0.28, *p* < 0.001), while social support was positively associated with both PCS (β = 0.32, *p* < 0.001) and MCS (β = 0.32, *p* < 0.001). For shidu parents, the proportion of mediation of social support between perceived stress and QOL was 36.85% for PCS and 29.45% for MCS, respectively.

**Conclusions:**

Perceived stress was associated with QOL and social support had a partially mediating effect between perceived stress and QOL in Chinese shidu parents. Low PCS and MCS of shidu parents highlight the need of timely developing interventions to reduce stress and reinforce social support to further improve their QOL.

## Background

“Loss-of-only-child family” refers to the family in which the only child died and the mother has passed her child-bearing age [1]. The parents who are unable to reproduce or do not foster other children are known as “shidu parents” in China [2]. The number of one-child families soared from 1979 to 2015 because of the one-child policy in China [3]. The annual number of deaths of only children is 76,000 based on the population census statistics in 2011 [4]. And according to the latest demographic statistics, the number of shidu parents in China will reach over 10 million by 2035 [5].

Shidu parents are a particularly vulnerable group in China, previous studies have shown that losing the only child can seriously damage the quality of life (QOL) of them both physically and mentally [6]. On the one hand, according to the investigation conducted on more than 1500 shidu families in 14 provinces of China published in 2013, there were more than half of them had incomes lower than the local standard of living, over 60% had chronic diseases and nearly half of the shidu families suffered from depression [7]. A recent study found that shidu parents were lonelier and indicated a lower level of cognitive functioning [8]. Shidu parents were at higher risk for insomnia, increasing medical visits, having cancer, and being hospitalized [9]. The higher death rate was found for shidu parents compared to parents with remaining children or parents who had given birth again. The 20-year longitudinal study conducted on 6284 Israeli parents who lost an adult child reported an evident increase in deaths of cancers [10]. On the other hand, losing the single child is an oppressive disaster for parents anywhere, but in China, the effect is exacerbated by Chinese culture, as parents are not only losing their single child but also losing their life's main emotional dependence and hope [11]. For parents, the dissolution of the attachment relationship with the child would elicit a series of emotional responses associated with loss, such as depression, anxiety, prolonged pain, and post-traumatic stress disorder (PTSD) [12]. These negative emotions can aggravate the grief of shidu parents so that seriously affecting their mental health. And shidu parents have been found to be more susceptible to suffering from mental disorders [13]. Thus, compared with parents who have living children, shidu parents face more difficulties in the society heavily relying on the bloodline and families [14], the physical health and mental health problems faced by shidu families have become increasingly prominent. However, to date, there were few studies have been conducted to report the QOL of Chinese shidu parents.

It has been affirmed that perceived stress was one of the risk factors for the poor QOL [15], while the loss of a child was considered one of the most stressful incidents in a parent’s life. In some researches on the psychological and social consequences of parents whose children died in natural or human-made disasters [6], it was shown that shidu parents suffered from multiple stresses including economic stress, social stress, and psychological stress. Firstly, the trauma of losing the single children in elderly age could lead many shidu parents to resign their jobs or retire prematurely, and some of the parents whose children died from diseases are often indebted because they have spent much money on medical care [16] so that they had to face the economic stress of life. Secondly, Chinese traditional culture emphasizes the continuity of the family line and attaches great importance to the culture of filial piety [17]. And there is a Chinese saying that “There are three unfilial acts, bearing no descendant is by far the most unforgivable” [18]. Therefore, given the stress to continue the family lineage, shidu parents would feel psychological distress because of the failure to live up to society’s expectations, while they were bitterly desperate due to the precipitate loss of their only child [19]. And shidu parents would be at high risk of suffering mental distress if the pressure is not relieved in time [20]. To our knowledge, there were few researches have been conducted to explore the impact of perceived stress on the QOL of Chinese shidu parents until now.

Previous study findings have also identified that poor QOL was associated with low social support [21]. Social support can be received from family members, friends, colleagues, and even strangers. High levels of social support lead to high levels of QOL. A cross-sectional study of older adults with osteoarthritis reported that the subjects were satisfied with their QOL because of the high levels of social support they received from families and friends, despite they were suffering from depression, comorbidity, pain, and functional limitation [22]. The previous study has shown that social support also played an important role in improving the QOL of psychiatric patients suffering from chronic schizophrenia [23]. Besides, a recent study showed a strong association between poorer social support and worse physical health [24]. Social isolation and lack of social support have been found to have an adverse effect on mental health [25]. The qualitative research on grief reactions of shidu parents suggested that the death of their single child necessarily came with social stigma and social withdrawal [26], which reduced their social support and thus their QOL. However, as far as we know, the relationship between social support and QOL of Chinese shidu parents also has been rarely conducted.

Some studies have also suggested that social support was negatively correlated with perceived stress. For instance, the significant effect of social support on reducing perceived stress among people living with HIV/AIDS (PLWHA) has been widely documented in both horizontal and longitudinal studies [27]. It has been shown that some psychosocial factors acted as buffers of the association between stress and QOL including mastery, social support, and coping [28]. A study of earthquake survivors suggests that social support can alleviate the negative effects on their QOL by protecting them from perceived stress [29]. And it has been widely demonstrated that social support can mediate the association between optimism and stress in individuals who have experienced a traumatic event [30], as well as between the higher levels of optimism and the increases in stress and depression of a sample of college students [31]. Similarly, a study confirmed that promote QOL by acting as a buffer against distress and cancer-related burden [32]. And the stress-buffering hypothesis supported the relationship of social support to the QOL depended on an individual’s level of perceived stress [33]. Based on the results of previous studies, we assume that social support may serve as a mediating variable between perceived stress and QOL among Chinese shidu parents.

The purpose of this study is to explore the relationships between perceived stress and QOL under the mediating effect of social support within a sample of Chinese shidu parents. The objectives are: [1] to excavate the QOL and the related factors of Chinese shidu parents; [2] to explore the relationship between perceived stress and QOL; [3] to estimate the effect of social support on QOL; [4] to examine the mediating role of social support between perceived stress and QOL.

## Methods

### Participants

From March 2017 to September 2017, the cross-sectional survey was conducted in five urban districts of Shenyang, the capital of Liaoning province in the Northeast of China. We randomly selected 15 communities in each district, screened all families with losing only children in the communities, and invited parents who met the inclusion criteria to be invited to participate in this study. The inclusion criteria were as follows: [1] were residing in the community for more than half a year; [2] lost the only child and have not reproduced or fostered other children; [3] were able to communicate clearly in Chinese. Among the 595 shidu parents who satisfied the inclusion criteria, 79 refused to take part in. The demographic characteristics were no significant difference between participants who agreed and those who refused to participate in the study. There were 516 questionnaires of respondents was collected, 14 were deleted from analysis because of data missing (more than 30%), which were completely random. Therefore, a total of 595 objects were initially involved in the survey, with 502 completing the questionnaire, an effective response rate of 84.37%.

### Procedure

The study was ethically approved by the Committee on Human Experimentation of China Medical University, and all the research processes met ethical standards. Participants were guaranteed confidentiality and had the right to withdraw at any time. The purposes and significance of the survey were explained to each participant before obtaining their written informed contents. With the help of the community family planning commissioners, parents who chose to participate were invited to the community office to fill out the self-report questionnaire, or they can choose to ask the investigators to visit them at home if they feel uncomfortable. The answers to the questionnaire were received through face-to-face interviews if the participants had difficulty filling them out on their own. In order to ensure the strictness and objectivity, as well as the smooth conduct of the survey, all community family planning commissioners took part in a training program. They were trained on research design scheme, field investigation method, and questionnaire recycling requirement, those who passed the training assessment would be the investigators of this survey. During the inquiries, the investigators were asked to avoid using suggestive language to ensure that each topic was the true idea of the participants. Before the formal investigation, the questionnaire was accurately checked and pre-survey was conducted to ensure that the participants could correctly understand the contents of the questionnaire.

### Measures

A polit study has been done before the formal investigation and the reliabilities and validities of all scales used in the present study have been examined. Scales with satisfactory reliabilities and validities would be applied in the formal survey. Basic demographic information including gender, age, employment, marital status, education level, chronic disease, annual household income, debt, marital satisfaction, the age and gender of the lost child, and whether they have a grandchild were collected by a self-report questionnaire. Investigators would help to explain in details if the participants had any questions about the questionnaire.

Perceived stress was measured using the Chinese version of the perceived stress scale-10 (PSS-10) [34], it consists of 4 positive items and 6 negative items to measure the degree to which life events are appraised as stressful. The PSS-10 can be applicable to any population subgroup, it has been found to be both reliable and valid [35]. In the present study, Cronbach’s alpha coefficient was 0.86 for the total scale.

The functional social support questionnaire (FSSQ) [36], consists of 8 five-category Likert items, which is a self-assessment tool created to measure individuals' perception of perceived FSS, and the higher score reflects the better social support. In this current sample, the Cronbach’s alpha for the FSSQ was 0.94.

We selected the MOS item short from health survey (SF-36), which was constructed to survey health status in the Medical Study [37]. The SF-36 measures eight QOL domains: Physical Functioning, Role Physical, Bodily Pain, General Health, Vitality, Social Functioning, Role Emotional, and Mental Health. These eight domains can be further summarized into two summary scores: the Physical Component Summary (PCS) and the Mental Component Summary (MCS) [38]. In this study, the Cronbach’s alpha coefficient was 0.95 for the total scale, the Cronbach’s alpha coefficient was 0.93 for the PCS scale and the Cronbach’s alpha coefficient was 0.89 for the MCS scale.

### Statistical analysis

The data were analyzed by the SPSS 19.0 Statistical Package. The simple statistics we performed to describe the distribution of demographic characteristics, such as means, standard deviations, and proportions. T-test and one-way ANOVA analyses were calculated to compare the PCS and the MCS scores of different participating groups. We used hierarchical multiple linear regression analysis to examine the influence of groups of independent variables on the PCS and MCS scores. Asymptotic and resampling strategies were conducted to explore the latent mediating effect of social support on the association between perceived stress and QOL. All tests were double-tailed, with *p* value < 0.05 indicating that the difference was statistically significant.

## Results

### Survey responses

The demographic characteristics and results of univariate analyses were shown in Table 1. Of the 502 study subjects, 205 (40.84%) were males and 297 (59.16%) were females. The mean age of the sample was 61.19 ± 6.71 years. Of the 502 study participants, results from the univariate analyses showed that age, chronic disease, employment, annual household income, debt, and marital satisfaction were statistically significant associated with PCS (*p* < 0.05). As well as the results of the univariate analysis manifested that chronic disease, employment, annual household income, debt, marital satisfaction and age of the child at death were associated with MCS (*p* < 0.05).

**Table 1:** Demographic characteristics of participants and result of univariate analysis

Variables	N (%)	PCS		*p*_1_	MCS		*p*_2_
		Mean	SD		Mean	SD	
*Gender*				0.61			0.62
Male	205 (40.84)	65.45	22.99		59.94	21.49	
Female	297 (59.16)	64.40	22.45		58.96	22.09	
*Age*				0.03			0.40
< 56	98 (19.52)	67.73	21.51		61.49	19.78	
56–66	303 (60.36)	65.57	22.62		59.37	22.24	
> 66	101 (20.12)	59.80	23.29		57.27	22.51	
*Marital status*				0.83			0.92
Single/divorced/widowed/separated	189 (37.65)	64.55	24.99		59.29	23.95	
Married/cohabited	313 (62.35)	65.00	21.16		59.49	20.49	
*Educational level*				0.49			0.55
Middle school or under	331 (65.94)	64.28	23.21		58.93	22.47	
Senior high school	141 (28.09)	65.15	21.51		59.52	20.70	
Undergraduate or above	30 (5.97)	69.42	21.87		63.45	19.99	
*Chronic disease*				< 0.001			< 0.001
Yes	280 (55.78)	56.12	22.87		53.36	21.98	
No	222 (44.22)	75.81	16.92		66.94	19.17	
*Employment*				0.003			0.02
Unemployment	411 (81.87)	63.32	23.19		58.29	22.39	
Part-time/full-time	85 (16.93)	71.44	19.20		64.28	18.78	
Missing	6 (1.20)						
*Annual household income (RMB)*				< 0.001			< 0.001
≤ 10,000	37 (7.37)	45.74	26.40		42.36	22.70	
10,001–30,000	229 (45.62)	63.13	22.41		57.89	22.42	
30,001–50,000	192 (38.25)	69.45	20.29		63.23	19.21	
> 50,000	44 (8.76)	69.63	21.39		64.47	21.55	
*Debt*				< 0.001			< 0.001
Yes	32 (6.37)	41.44	23.22		42.46	21.90	
No	467 (93.03)	66.51	21.77		60.64	21.38	
Missing	3 (0.60)						
*Marital satisfaction*				0.002			< 0.001
Unsatisfactory	85 (16.93)	58.37	25.53		51.79	22.49	
Satisfactory	409 (81.47)	66.54	21.59		61.18	21.03	
Missing	8 (1.60)						
*Gender of the lost child*				0.35			0.28
Male	325 (64.74)	65.44	22.00		60.02	20.94	
Female	173 (34.46)	63.43	23.96		57.78	23.49	
Missing	4 (0.80)						
*Reason of the child’s death*				0.94			0.49
Accident	131 (26.10)	64.21	23.63		58.66	22.25	
Acute disease	198 (39.44)	64.69	22.01		59.71	21.68	
Chronic disease	150 (29.88)	65.21	22.43		58.76	21.57	
Mental illness	19 (3.78)	67.55	26.15		66.70	22.75	
Missing	4 (0.80)						
*Age of the child at death*				0.10			0.03
≤ 15 year old	78 (15.54)	65.62	24.20		60.03	23.12	
16–25 year old	191 (38.04)	67.04	21.61		62.31	20.32	
≥ 26 year old	228 (45.42)	62.35	22.87		56.59	22.30	
Missing	5 (1.00)						
*Time since the child’s death*				0.24			0.07
≤ 5	202 (40.24)	62.72	22.61		56.73	21.91	
6–15	180 (35.85)	66.43	22.16		61.88	21.32	
≥ 16	115 (22.91)	65.85	23.49		59.79	22.17	
Missing	5 (1.00)						
*Whether they have a grandchild*				0.10			0.16
Yes	60 (11.95)	60.33	21.15		55.64	21.53	
No	423 (84.27)	65.50	22.87		59.89	21.85	
Missing	19 (3.78)						

*p*_1_ shows the difference of PCS scores in different groups created with demographic data.* p*_2_ shows the difference of MCS scores in different groups created with demographic data

### Correlations between the study variables

Correlations among perceived stress, social support, and QOL (PCS and MCS) were shown in Table 2. Both perceived stress and social support were significantly associated with PCS and MCS. Perceived stress was negatively associated with PCS (r =  − 0.46, *p* < 0.01) and MCS (r =  − 0.49, *p* < 0.01). Social support was positively associated with both PCS (r = 0.50, *p* < 0.01) and MCS (r = 0.49, *p* < 0.01). Perceived stress was negatively associated with social support (r =  − 0.42, *p* < 0.01).

**Table 2:** Correlation coefficients among perceived stress, social support and QOL (PCS and MCS)

Variables	Mean (SD)	1	2	3	4
1. Perceived stress	19.67(5.51)	1			
2. Social support	23.58(7.70)	− 0.42*	1		
3. PCS	64.83(22.66)	− 0.46*	0.50*	1	
4. MCS	59.36(21.83)	− 0.49*	0.49*	0.83*	1

^*^p < 0.01(two tailed)

### Hierarchical multiple linear regression results

The SF-36 QOL score consisted of PCS score and MCS score was used as the dependent variable for hierarchical regression analysis, and the results are presented in the tables.

As shown in Table 3, chronic disease, employment, annual household income, debt, and marital satisfaction explained 27.50% of the PCS change (*p* < 0.001). Perceived stress and social support totally contributed to 18.10% of the variance in PCS (*p* < 0.001). The results indicating that perceived stress had a negative effect on PCS (β =  − 0.21, *p* < 0.001), and the social support was positively associated with PCS (β = 0.32, *p* < 0.001).

**Table 3:** Hierarchical multiple linear regression analysis result of PCS

Variables	PCS		
	Step 1(β)	Step 2(β)	Step 3(β)
Age	− 0.05	− 0.06	− 0.06
Chronic disease	− 0.38***	− 0.32***	− 0.29***
Employment	0.09*	0.05	0.06
Annual household income (yuan)	0.16***	0.14**	0.10**
debt	− 0.19***	− 0.13**	− 0.12**
Marital satisfaction	0.08	0.03	0.01
Perceived stress		− 0.34***	− 0.21***
Social support			0.32***
F	30.27***	40.87***	49.94***
R^2^	0.28	0.37	0.46
△R^2^	0.28	0.10	0.08

^*^p < 0.05, **p < 0.01, ***p < 0.001

Similarly, as shown in Table 4, chronic disease, employment, annual household income, debt, marital satisfaction, and age of the child at death explained 17.20% of the MCS change (*p* < 0.001). Perceived stress and social support totally contributed to 22.20% of the variance in MCS (*p* < 0.001). The results indicating that perceived stress had a negative effect on MCS (β =  − 0.28, *p* < 0.001), and the social support was positively associated with MCS (β = 0.32, *p* < 0.001).

**Table 4:** Hierarchical multiple linear regression analysis result of MCS

Variables	MCS		
	Step 1(β)	Step 2(β)	Step 3(β)
Chronic disease	− 0.28***	− 0.21***	− 0.18***
Employment	0.09*	0.05	0.06
Annual household income (yuan)	0.14**	0.11	0.07
debt	− 0.13**	− 0.07	− 0.05
Marital satisfaction	0.12**	0.06	0.04
Age of the child at death	− 0.03	− 0.05	− 0.06
Perceived stress		− 0.41***	− 0.28***
Social support			0.32***
F	16.39***	31.33***	38.36***
R^2^	0.17	0.32	0.39
△R^2^	0.17	0.15	0.08

^*^p < 0.05, **p < 0.01, ***p < 0.001

### Mediation analyses results

Asymptotic and resampling strategies were conducted to explore that social support as a possible mediating role on the relationship between perceived stress and QOL, and calculated the 95% confidence interval, all the results were shown in Table 5. For both PCS and MCS, the total effect of perceived stress on the PCS and MCS (coefficient c) was preliminarily assessed. Next, the indirect effect of perceived stress on PCS and MCS via social support was examined (coefficient a*b). Finally, the direct effect of perceived stress on PCS and MCS (coefficient c’) was still significant statistically when social support was entered in the effect as a mediator, and BCa95%CI didn't include 0. To examine the size of the mediator's influence, we used the formula (a*b)/c to calculate the proportion of social support mediating in the total effect of perceived stress on the QOL, and the results showed that the proportion of mediation of social support was 36.85% for PCS and 29.45% for MCS.

**Table 5:** The mediating role of perceived stress between social support and QOL

	Mediators	c	a	b	c′	a*b(BCa95%CI)
PCS	Social support	− 0.33*	− 0.38*	0.32*	− 0.21*	− 0.12 (− 0.16, − 0.09)
MCS	Social support	− 0.40*	− 0.38*	0.31*	− 0.28*	− 0.12 (− 0.16, − 0.08)

^*^p < 0.001

Thus, in this study, it was proven that social support had a partially mediating effect between perceived stress and QOL.

## Discussion

In this study, the mean SF-36 score of PCS was 64.83 ± 22.66 and the mean score of MCS was 59.36 ± 21.83. It was measured by the general population of 6 Chinese cities that the score on PCS was 77.54 ± 15.96 and on MCS was 71.29 ± 17.86 in 2011 [39]. Therefore, the QOL score of shidu parents was much lower than that of the general population in China. The loss of an only child often meant the loss of their primary source of health care, the end of family continuity, and a significant hazard to personal happiness [40]. For Chinese shidu parents, certain unique factors impacted their physical and mental health. The first was the cultural stigma associated with children’s death. Chinese culture regarding the death of a child as a symbol of bad luck and a taboo subject [17], communicating with shidu parents about the child’s death was often provoking and can be painful and insulting [2]. Another factor was that the one-child policy made shidu parents unique. Research indicated that one-child parents were more probable to rank their children as the most vital aspect of their lives compared with multiple-child parents [41]. Additionally, family care was a foundational way of caring in China. And as primary caretakers of the old people, adult children had the accountability to meet the mental health needs of parents [42]. Thus, the death of the only child meant not only the great sorrow and physical or mental damage, but also the termination of expected financial and social support both culturally and legally from the child for the parents.

Our results indicated that having chronic diseases was a risk factor for both PCS and MCS. Shidu parents had a higher incidence of chronic diseases, it was reported that more than 60% of those surveyed had chronic diseases in an investigation [7]. In addition, lower incomes and being in debt were found to be associated with a lower PCS score of the shidu parents. As they grow older, shidu parents face not only declining health but also financial difficulties. The income of some old parents was not stable, and the cost of aged care, medical care, funeral, and other expenses have been increased significantly [43]. When the only child died, the old parents virtually had no other reliable sources for aged care, and shidu parents’ rights to subsistence would be at risk without a stable source of income [14]. According to a survey in 2013, more than 60% of the shidu families examined said they had financial difficulties [44]. In the present study, we found that parents with debt had worse QOL than those without debt.

The results confirmed the conspicuous negative correlation between perceived stress and QOL, which was consistent with previous studies [45, 46]. Chinese shidu parents were burdened with stress both physically and mentally. On the one hand, "Raising a child for old age" is an inherent concept in Chinese traditional culture. The old parents need to live on their children, and it is a tradition of Chinese to support parents since ancient times. It was difficult to maintain families for shidu parents who were suffering from various difficulties including economic, spirit, pension, medical treatment of a serious disease [47]. But they did not have alimony given by their child, not to mention the other supports. On the other hand, Chinese culture is collectivist, regarding the interests of the family as the most important [18]. For Chinese parents who have lost their only child, “shidu” meant not only the loss of their only child but also the loss of the hope of having offspring and carrying on the family line, which was seen as the responsibility to ancestors in familism culture [17]. The unrealized and unfulfilled duty could cause the psychological pressure of shidu parents. And it was harmful to shidu parents’ QOL to face the stress both physically and mentally. The previous study has reported that there was a negative connection between stress with QOL [48], the more stress was related to lower levels of physical and mental health.

What's more, this study supported that social support was positively associated with QOL. It was shown that shidu parents received less social support from friends or families than they expected [49]. For bereaved parents, the loss of their child could lead to a decline in social status, which produced the loss of interest in social life [50]. After the children’s death, shidu parents not only suffered from social isolation but also designedly isolated themselves from social relationships [51]. There was a relationship between poor social support and lower levels of QOL. For example, the study in a sample of American women indicated that poor social support had a significant effect on poor functional statuses such as physical health problems and role limitations [52]. Previous studies have also found that lower social support was associated with increased blood pressure [53], hyperlipemia [54], and lower nutrient intake [55]. Previous research has suggested that social support was a pivotal factor in helping shidu parents solve their problems of adjustment and deal with the loss of children [56]. Thus, increasing social support is conducive to improving the QOL of shidu parents.

As expected, the effect of social support on mediating the relationship between perceived stress and QOL in 502 shidu parents was examined. Our results indicated that social support was positively associated with QOL and negatively associated with perceived stress, and as a mediator between perceived stress and QOL, this finding was in accord with previous studies [21, 46]. Previous researches had consistently shown that social support can offer a psychological buffer against life stress, anxiety, and depression [57]. And social support mediated stress and greater stress was associated with decreases in social support in an investigation [58]. The study among parents of disabled children supported that social support mediated parenting stress and QOL, with the higher the parenting stress, the lower social support, and the worse QOL [59]. Research of PLWHA has been proved that high stress had a negative impact on QOL directly, and then affected the QOL by decreasing the level of social support [60]. Therefore, it should be taken seriously to improve the QOL by reducing the perceived stress of shidu parents, because stress can directly affect QOL and indirectly through social support.

Overall, shidu was a particular social problem in Chinese society after the decades of implementation of the one-child policy. Children were the primary source of joy, hope, and well-being in life for all parents, and it would trigger the most devastating grief and a great deal of stress for parents to lose a beloved child [61]. Compared with parents who have not experienced this loss, shidu parents were at higher risk for developing mental health problems and physical health constraints. The death of a child led to a markedly higher level of unresolved grief, which could worsen physical and mental health based on the researches conducted with the bereaved parents. Therefore, the government and society would attach importance to improve the medical security service system for this unique group of shidu parents or provide shidu parents with home care within their means. Previous findings supported that furnishing shidu people with the strategies to cope with stress effectively may promote their QOL [6]. It was recommended that stress management would be incorporated into interventions to improve the QOL for shidu parents. The essential thing to shidu parents to provide them with systematic and culturally sensitive psychological assistance and mental health services [2]. For instance, it could be more conducive to reducing stress by providing exclusive psychological counseling channels and organizing activities to promote peer communication for shidu parents. And developing appropriate social support can be taken into account as a vital step of psychological intervention strategies to improve the QOL for bereaved parents. Besides economic support, social support in the manner of grief counseling is also needed to prevent a decline in mental health [62]. The researchers proposed that subjects with a certain occupation would have a larger social network and receive more social support from others [63]. Hence, the government can introduce corresponding policies to encourage shidu parents to get back jobs and participate in more social activities, which not only improve their economic situation but also reduce their social isolation and improve their QOL.

There were several limitations to the study. Firstly, the actual score of QOL may be lower than the results reported here, because the participants were selected by the certain criteria voluntarily and some shidu parents still cannot accept the fact that they choose not to take part in the survey, so the sampling method may not representative of all shidu parents in China. Secondly, this study concentrated mainly on the relationships among perceived stress, social support, and QOL, more studies are needed to explore the influence factors for the QOL of shidu parents. Thirdly, more investigations should be carried out to address the factors that make up the differences in shidu parents’ QOL between different cultural backgrounds. Finally, the present study was a cross-sectional study and our results cannot be used to build the causal relationships, and future researches using a longitude design care needed to validate the findings.

## Conclusions

The QOL of shidu parents was lower than the general population in China, they had both physical and mental health problems. Losing the only child was the most traumatic incident for shidu parents, which was a grievous and unique issue in Chinese society result from the one-child policy. Results of the present study supported that perceived stress negatively influenced QOL and social support had a partially mediating effect between perceived stress and QOL. We should pay more attention to the physical and mental health of shidu parents and provide them with more conveniences and psychological counseling in the future. Considering the increase in the number of shidu parents and the reduction of their QOL as well as the stress they suffer, more social support should be directed to improve their health both physically and mentally. Meanwhile, to better their life quality, more health care-related decision is desired to address those problems and challenges the shidu parents are facing.

## Data Availability

The datasets used and/or analyzed during the current study are available from the corresponding author on reasonable request.

## Abbreviations

QOL: Quality of life
PTSD: Post-traumatic stress disorder
PLWHA: People living with HIV/AIDS
PSS: Perceived stress scale-10
FSSQ: Functional social support questionnaire
PCS: Physical component summary
MCS: Mental component summary
ANOVA: Analysis of variance
